# Multi-country IgE reactivity determination of isoforms of the *Blomia tropicalis* mite allergen Blo t 2 revealed a potential biomarker of asthma severity

**DOI:** 10.3389/fimmu.2026.1815807

**Published:** 2026-04-07

**Authors:** Juan R. Urrego, Lorenz Aglas, Sara Huber, Emília M. M. A. Belitardo, Peter Briza, Álvaro A. Cruz, Luis F. Salazar-Garcés, Philip J. Cooper, Josefina Zakzuk, Luis Caraballo, Carina S. Pinheiro, Neuza M. Alcântara-Neves, Fatima Ferreira, Eduardo S. da Silva

**Affiliations:** 1 Laboratory of Allergology and Acarology (LAA), Institute of Health Sciences, Federal University of Bahia, Salvador, Brazil; 2 Post-Graduate Program in Biotechnology of the Northeast Biotechnology Network (RENORBIO), João Pessoa, Brazil; 3 Department of Biosciences, Paris-Lodron University of Salzburg, Salzburg, Austria; 4 Department of Pharmacy, University of Cartagena, Cartagena, Colombia; 5 Human Microbiome (HUMI) Research Program, Faculty of Medicine, University of Helsinki, Helsinki, Finland; 6 Post-Graduate Program in Immunology, Institute of Health Sciences, Federal University of Bahia, Salvador, Brazil; 7 ProAR Foundation and Federal University of Bahia, Salvador, Brazil; 8 Faculty of Health Sciences, Technical University of Ambato, Ambato, Ecuador; 9 Institute of Infection and Immunity, St George’s University of London, London, United Kingdom; 10 School of Medicine, International University of Ecuador, Quito, Ecuador; 11 Institute for Immunological Research, University of Cartagena, Cartagena, Colombia

**Keywords:** allergy, group 2 mite allergens, mite, recombinant allergen, severe asthma

## Abstract

**Background:**

*Blomia tropicalis* is an important source of inhalant allergens in Southeast Asia and Latin America. Previous proteomics identified multiple Blo t 2 isoforms, yet their clinical relevance to Latin American populations and association with asthma phenotypes remain a priority in the field.

**Objective:**

To produce and purify the two recombinant isoforms rBlo t 2.2 and rBlo t 2.5 and physicochemically characterize them.

**Methods:**

Isoforms were expressed in *E. coli* Shuffle T7 and purified via cation-exchange chromatography. Structural features were verified by mass spectrometry and Fourier-transform infrared spectroscopy. Further, proteolytic stability and LPS-binding activity were determined. IgE-reactivity and allergenic activity were evaluated by Western blot, ELISA and mediator release assays. Sensitization patterns were analyzed in serum samples of *Blomia tropicalis*-allergic individuals with and without asthma from Brazil, Colombia, and Ecuador.

**Results:**

Both isoforms were correctly folded with proper disulfide bonds and exhibited high stability against endolysosomal proteases. Recombinant Blo t 2.2 lacked specific LPS-binding activity and displayed low cross-reactivity with rDer p 2. Across the three countries, the average sensitization rate to rBlo t 2.2 was 50%. Specifically, sensitization was observed in 47% of Brazilian teenagers and 52% of adults, 53% of Colombian individuals, and 48% of Ecuadorian children and teenagers. Sera from the Ecuadorian study exhibited the highest IgE reactivity. The rBlo t 2.2 isoform displayed significantly higher IgE-binding than rBlo t 2.5 in Brazil. Sensitization to rBlo t 2.2 was significantly more frequent in severe asthma patients than in mild asthma and rhinitis patients.

**Conclusion:**

rBlo t 2.2 is a stable, mid-tier to major isoform and a candidate biomarker for severe asthma in Brazil. Its structural and immunological characteristics support its inclusion in molecular diagnostic panels. However further investigations with larger sample sizes and application of multivariate analyses are still warranted to confirm our findings.

## 1 Introduction

Allergic illnesses are considered a public health burden in several countries worldwide. Among the various manifestations, asthma is a highly prevalent chronic non-communicable disease, affecting an estimated 3,340 cases per 100,000 individuals and projections suggest that the global incidence will remain elevated at least until 2050 (1–3). Allergic asthma is triggered by inhalant allergens derived from pollen, animal dander, or house dust mites (HDM) (4, 5).

HDM are considered the main source of indoor allergens, causing a variety of allergic symptoms worldwide (6, 7). Although classified as a storage mite, *Blomia tropicalis* is also found in house dust and can be considered a species of HDM (8, 9), sensitizing 55% to 93% of allergic patients in different regions of the globe (10–12). Its clinical relevance has been clearly shown in tropical and subtropical regions (9, 11–16). Apart from the morphological similarities of *B. tropicalis* to other allergenic mites such as those of the genus *Dermatophagoides*, the amino acid sequence identity of homologous proteins between these mites is rather low; about 40% (12, 17).

To date, 26 different *B. tropicalis* allergens have been named by the International Union of Immunological Societies (IUIS) nomenclature subcommittee (12, 18–23). Blo t 5 and Blo t 21 are well-known major allergens, reaching up to 90% IgE sensitization frequency among *B. tropicalis* sensitized individuals in some countries (12–14, 16, 19, 24). However, only in recent years has the clinical importance of Blo t 2 been established (19, 25–27). Group 2 HDM allergens display structural homology to myeloid differentiation-2 (MD-2), an immunoglobulin-like fold protein with two planes of β-sheets that fold to form a large internal hydrophobic cavity which binds to lipopolysaccharide (LPS) (28, 29). Group 2 mite allergens are major allergens in *Dermatophagoides farinae/pteronyssinus*, other glycyphagid mites like *Lepidoglyphus destructor* (16, 30–33) and *B. tropicalis* (27).

While recent studies in Colombia, Singapore, and Cuba have provided valuable insights into Blo t 2 sensitization (25–27), some knowledge gaps remain. Previous research has lacked both a rigorous physicochemical analysis of recombinant isoforms and a multicenter comparison across different populations. Consequently, the relationship between specific Blo t 2 isoforms and asthma severity remains poorly defined, necessitating a more integrated evaluation across diverse geographical settings. Furthermore, our group’s previous proteomic analysis identified several Blo t 2 isoforms as the most abundant among *B. tropicalis* allergens (34). Given these considerations, the aims of this study were: (i) to physicochemically characterize two isoforms of Blo t 2 (the most abundant in the proteomic study), termed here rBlo t 2.2 and rBlo t 2.5 (GenBank IDs, ABG76186 and AAQ73483, respectively) (ii) to determine Blo t 2 sensitization frequency in Brazil, Colombia and Ecuador; and (iii) to evaluate whether Blo t 2 can be used as a potential biomarker for severe allergic asthma.

## 2 Methods

### 2.1 Design and synthesis of harmonized rBlo t 2 isoform sequences.

Blo t 2.2 (ABG76186) and Blo t 2.5 (AAQ73483) sequences were modified for optimal *E. coli* heterologous expression via codon harmonization (http://www.kazusa.or.jp/codon/) (35). Blo t 2.2 and Blo t 2.5 sequences have been acknowledged by the IUIS under isoallergen IDs Blo t 2.0105 and Blo t 2.0101, respectively (https://www.allergen.org/index.php). Harmonized sequences were synthesized by ATG:biosynthetics (Germany) in the pET-30a(+) cloning vector. rDer p 2 was produced in-house as previously described (36).

### 2.2 Expression and purification of rBlo t 2 isoforms

Cloning vectors were co-transformed into *E. coli* SHuffle T7 (New England Biolabs, USA) with chaperone plasmid set pG-KJE8^®^ (Takara Bio Inc, Japan). Protein expression (250 mL LB medium) was induced with 0.4 mM isopropyl β-D-1-thiogalactopyranoside at an OD_600_ of 0.6. and incubated for 5 hours at 37 °C (200 rpm). Chaperones were induced at OD_600_ 0.3 with 0.5 mg/mL arabinose and 5 ng/mL tetracycline. The bacterial pellet was resuspended in lysis buffer (50 mM Tris-HCl pH 8.5, 0.5 M urea), lysed via freeze/thaw cycles, and homogenized using an Ultra-Turrax T25^®^ (IKA, USA). After DNase I treatment and centrifugation (13,000 g, 20 min), chaperones and soluble proteins were precipitated with acetic acid (pH 4) overnight. The supernatant was purified via HiTrap SP FF cation exchange chromatography (Cytiva, Sweden) using a gradient of 50 mM Tris pH 4.0, 0.5 M urea, and 0.5 M NaCl (36). Purification was verified by 15% sodium dodecyl sulfate-polyacrylamide gel electrophoresis (SDS-PAGE).

### 2.3 Structural characterization of purified rBlo t 2 isoforms

Identity and primary structure were assessed via mass spectrometry (MS) and amino acid analysis (AAA). AAA was performed using the PicoTag™ method (Waters, Milford, MA, USA) on an HP1100 HPLC system (Hewlett-Packard, San Jose, CA, USA) equipped with a 3.9 x 150 mm Nova-pak C18 column (Waters). Hydrolyzed amino acid peaks were quantified at 254 nm by peak area comparison to Amino Acid Standard H (Pierce, Rockford, IL, USA) (37–41). MS analysis was performed using a quadrupole time-of-flight mass spectrometer with electrospray ionization (ESI-QTOF-MS; Waters Corp), as previously detailed (37–41).

Secondary structural elements were verified by Fourier-transform infrared spectroscopy (FTIR), following previously described protocols (37–41). Briefly, an AquaSpec transmission cell adapted to a Tensor II Confocheck FTIR system (Bruker Optics Inc., Billerica, MA, USA) was used to obtain the infrared spectra of the variants, at 25 °C, with a data acquisition time of 100 seconds, and spectral resolution of 4 cm^-1^. Using Savitzky–Golay algorithm and 25 smoothing points, the second derivatives were calculated from the absorbance spectra and vector-normalized on the Amide I band (37–41).

### 2.4 Endolysosomal degradation assay

Assays were performed by incubating rBlo t 2 isoforms with microsome-derived endolysosomal extracts from JAWS II cells (41, 42). In brief, 0.25 µg/µL of the protein was incubated with 0.4 μg/μL of the isolated microsomal proteins in 100 mM citrate buffer (pH 4.8) and 2 mM DTT. Reactions at 37 °C for up to 48 h were stopped by boiling and evaluated by flatbed scanner densitometry of GelCode^®^ Blue-stained SDS-PAGE gels.

### 2.5 Surface acoustic wave measurement

Due to the high structural similarity between the two isoforms, the LPS-binding activity was characterized exclusively for Blo t 2.2 using SAW technology, which has been previously been employed to study LPS binding to antimicrobial peptides and allergens (43, 44). The protein purification protocol included an acidic precipitation step, which has previously been shown to significantly reduce endotoxin levels in recombinant protein samples (45). Since endotoxin contaminations might interfere with the determination of LPS-binding activity of Blo t 2.2 by masking potential interactions, we determined the endotoxin concentration in Blo t 2.2 using the Pierce™ Chromogenic Endotoxin Quant Kit (Thermo Scientific, Waltham, MA, USA). The measured endotoxin concentration was 3x10–^4^ EU/µg, which is well below the established threshold for recombinant proteins (<1 EU/µg of protein) (46, 47). Following this verification, rBlo t 2.2 was immobilized on a SAW Chip CM-Dextran 3D using the Sam^®^ 5BLUE biosensor (Nanotemper, Germany). To determine the affinity constant (*K_D_*), serial dilution of *Escherichia coli* O111:B4 LPS (250- 12.5 μM) was injected into the buffer stream. The endotoxin concentration measured in Blo t 2.2 was negligible compared to the LPS concentrations used in the assay (ca. 10^6^ EU/mL LPS for lowest concentration), thus, we could exclude pre-associated endotoxin as parameter interfering with the assay´s performance. Between LPS sample injections, the allergen surface of the chip was regenerated to a baseline level using a 10 mM citric acid buffer pH 2.8. All SAW experiments were performed using endotoxin-free buffers. Kinetic analysis was performed with TraceDrawer 1.7 software (43, 48). Controls included Kdo2-lipid A, which is a Toll-like receptor 4 (TLR4) activator and ANS (hydrophobic patch binder) (49). To validate results, 25 µM biotinylated LPS-EB (Invivogen, USA) was immobilized on a SAW streptavidin chip, and rBlo t 2.2 was titrated (0–50 µM). Recombinant Bet v 1 served as a negative control (43).

### 2.6 Human sera samples

This multi-country study utilized 233 sera from children, teenagers, and adults across three countries. This broad age distribution was selected to investigate potential age-dependent sensitization patterns and to validate the geographical consistency of IgE reactivity to Blo t 2 isoforms. By including participants across various life stages, we aimed to account for the clinical diversity of Blo t sensitization regardless of age or regional environmental differences. Inclusion criteria included a clinical history of allergy, positive skin prick tests (SPT > 3 mm) to *B. tropicalis* extract (BtE), and specific IgE (sIgE) >0.70 kU/L (ImmunoCAP^®^ d201) (50, 51). The presence of sIgE against BtE in Ecuadorian samples were confirmed via in-house ELISA, as detailed previously (52).

Regarding the Colombian samples, 46 sera from individuals (3 to 82 years of age) were used in the present study and comprised of 36 allergic and 10 non-allergic sera. Out of the 57 children and teenagers (6 to 16 years of age) sera from Ecuador, 44 were included as allergic and 13 as non-allergic. Regarding the Brazilian serum samples, either sera of 40 teenagers (12 to 18 years of age, 30 were allergic and 10 non-allergic) enrolled in the SCAALA (Social Change in Asthma and Allergy in Latin America) program (53, 54) were included for a initial screening of IgE reactions against the two Blo t 2 isoforms and rDer p 2; or 90 adult subjects (21 to 40 years of age) attending the Bahia State Program for the Control of Asthma and Allergic Rhinitis (ProAR) Foundation (55, 56) were included. Among the latter, 64 were allergic and 26 non-allergic.

Informed written consent was obtained from all participants or legal guardians. Protocols were approved by the Ethics Committees of the Federal University of Bahia (ProAR, CAAE 45376814.0.0000.5577; SCAALA, 120.616), Hospital Pedro Vicente Maldonado and Pontificia Universidad Catolica del Ecuador (Ecuador, 9-12–04 and CBE-007-010), and the University of Cartagena (Colombia).

### 2.7 Asthma phenotypes

ProAR donors were classified into allergic rhinitis (AR), mild asthma (MA), or severe asthma (SA) using GINA criteria and an allergist audit (57). Patients with other chronic respiratory diseases were excluded (55, 56). For SA patients, inhaled and systemic corticosteroid usage was categorized as intermediate (800–1,600 μg budesonide-equivalent) or high (>1,600 μg) (58–60).

### 2.8 Immunoblotting rBlo t 2 isoforms

For immunoblot analysis, recombinant isoforms (10 μg/well) or BtE (25 μg/well) were transferred onto nitrocellulose membranes (Whatman, UK). To prevent nonspecific binding, membranes were blocked with 10% (w/v) bovine serum albumin (BSA) in Tris-buffered saline with 0.05% Tween 20 (TBS-T). The membranes were then incubated overnight at 4 °C with a 1:10 dilution of a sera pool from 12 allergic patients, randomly selected from the SCAALA study; this choice was made based on the higher volume of available sera in the SCAALA study. IgE reactivity was detected using an AP-conjugated mouse anti-human IgE antibody (1:10,000) for 2 hours at room temperature, and developed with nitroblue tetrazolium and 5-bromo-4-chloro-3-indolyl-phosphate substrates (40, 41).

### 2.9 IgE reactivity of rBlo t 2 isoforms

Initial screening of 30 Brazilian teenagers was performed by ELISA (Nunc MaxiSorp^®^) coated with rBlo t 2.2, rBlo t 2.5, or rDer p 2 (2.5 μg/mL) (36). Plates were blocked with TBS-T-BSA, incubated with sera (1:10), and detected with AP-conjugated anti-human IgE (1:4,000) using pNPP substrate (405 nm). Subsequently, clinical significance was examined in the full Brazilian, Colombian, and Ecuadorian samples (n = 144, only allergic to *B. tropicalis*). This protocol was modified to use biotin-conjugated monoclonal anti-human IgE (1:1,000), streptavidin-HRP (1:2,000), and 3,3’,5,5’-tetramethylbenzidine substrate, stopped with 4 M sulfuric acid (450 nm). The IgE cutoff was defined as the mean plus 3 SD of non-allergic sera (26, 41). For the initial screening using sera from the SCAALA study, 10 non-allergic subjects were utilized to determine the cutoff, while 49 non-allergic subjects were used to establish the cutoff for the multi-country reactivity evaluation.

### 2.10 Mediator release assay

RBL-2H3 cells expressing the human Fc-ϵRI alpha-chain were sensitized with sera (1:20) from four allergic patients (61). Crosslinking was induced by serial dilutions (10^4^ to 10–^4^ ng/mL) of rBlo t 2.2, rBlo t 2.5, and rDer p 2. β-hexosaminidase release was measured and expressed as a percentage of total enzyme secretion. Half-maximal release was calculated as previously reported (41).

### 2.11 Inhibition ELISA

Based on results from the preliminary IgE binding and mediator release assays, rBlo t 2.2 was selected as the representative isoform for subsequent cross-inhibition assays, performed according to established protocols (40, 62). Sera from 6 randomly selected teenagers were used in the assay; all were IgE reactive to rBlo t 2.2 and rDer p 2 (SCAALA study). The inhibition signal (10^-3–^10^2^ ng/mL of allergen inhibitor) was quantified via comparison with a non-inhibited serum pool. Percentage of inhibition was calculated by the formula: I% = (OD serum without inhibition - OD inhibited serum)/OD without inhibition × 100%. Where “I%” corresponds to percent inhibition and “OD”, at optical density.

### 2.12 Statistical analyses

Data were processed in GraphPad Prism 8.4.3 (GraphPad Software, San Diego, CA, USA). Normality was verified via the Shapiro-Wilk test. Comparisons between three groups utilized one-way ANOVA with Bonferroni’s *post hoc* test or Kruskal-Wallis with Dunn’s *post hoc* test. Paired groups were compared using paired t tests or Wilcoxon signed-rank tests. Pearson’s or Spearman’s coefficients were determined for correlations between IgE reactivity and BtE data (52). Fisher’s exact test confirmed differences in sensitization rates across AR, MA, and SA phenotypes and steroid usage (63). For the SA groups, data were stratified by quartiles: the 25^th^ percentile (0.220; intermediate/low), 50^th^ percentile (0.975; high), and 75^th^ percentile (1.539; highly reactive). To ensure statistical power, the high and highly reactive categories were merged for comparison against corticosteroid usage.

## 3 Results

### 3.1 Structural and functional characterization of Blo t 2 isoforms

**Figure 1:**
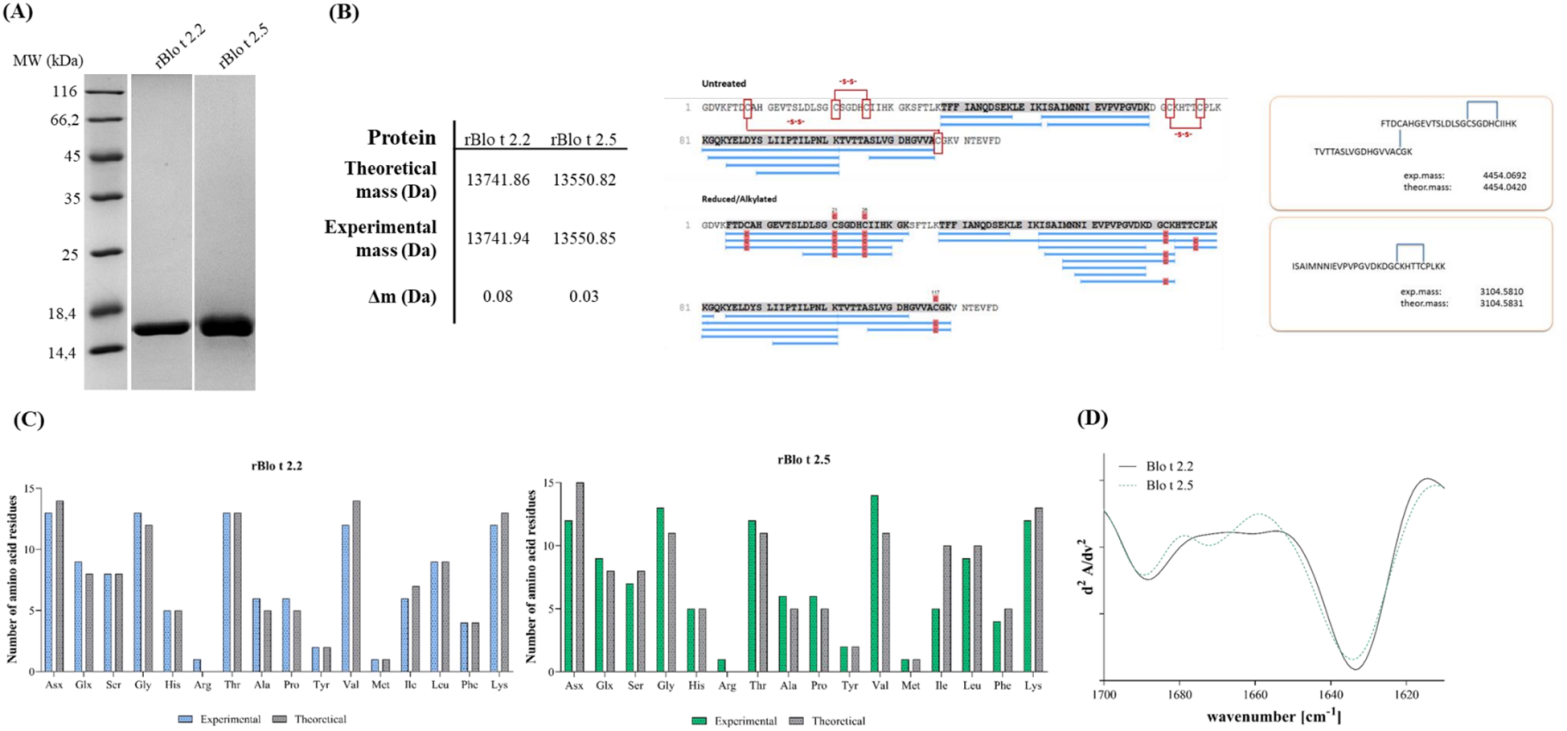
Structural characterization of rBlo t 2 isoforms. **(A)** 15% SDS-PAGE analysis of representative rBlo t 2.2 and rBlo t 2.5 fractions purified via cation exchange chromatography. **(B)** Mass spectrometry confirmation of protein identity and disulfide bond formation. The sequence coverage map for rBlo t 2.2 is shown under untreated and reducing conditions (center). Disulfide bond mapping under nonreducing conditions (right) displays a complex formed by two peptides (upper panel) and a single peptide sequence (lower panel), with experimental and theoretical masses indicated. **(C)** Primary structural elements were verified by amino acid analysis. **(D)** Secondary structural elements were analyzed Fourier-transform infrared spectroscopy.

The expressed rBlo t 2 isoforms were purified to homogeneity under nondenaturing conditions via cation exchange chromatography (**Figure 1A**). The molecular weights, determined by measuring the intact mass by MS, were 13,741.94 Da for rBlo t 2.2. and 13,550.85 Da for rBlo t 2.5 and in line with their corresponding theoretical values (**Figure 1B**). Moreover, tryptic digestions, performed either under non-reducing conditions or under reducing and alkylating conditions, revealed that in the untreated sample no linear peptides at the expected locations of the cysteine residues in rBlo t 2.2 were found in comparison to reduced, alkylated rBlo t 2.2, where a nice coverage of peptides was observed. Identical results were found for rBlo t 2.5 (data not shown). These data confirm the structure of the recombinant Blo t 2 isoforms based on the correct formation of disulfide bonds, obtained during heterologous expression using the *E. coli* strain SHuffle T7.

**Figure 2:**
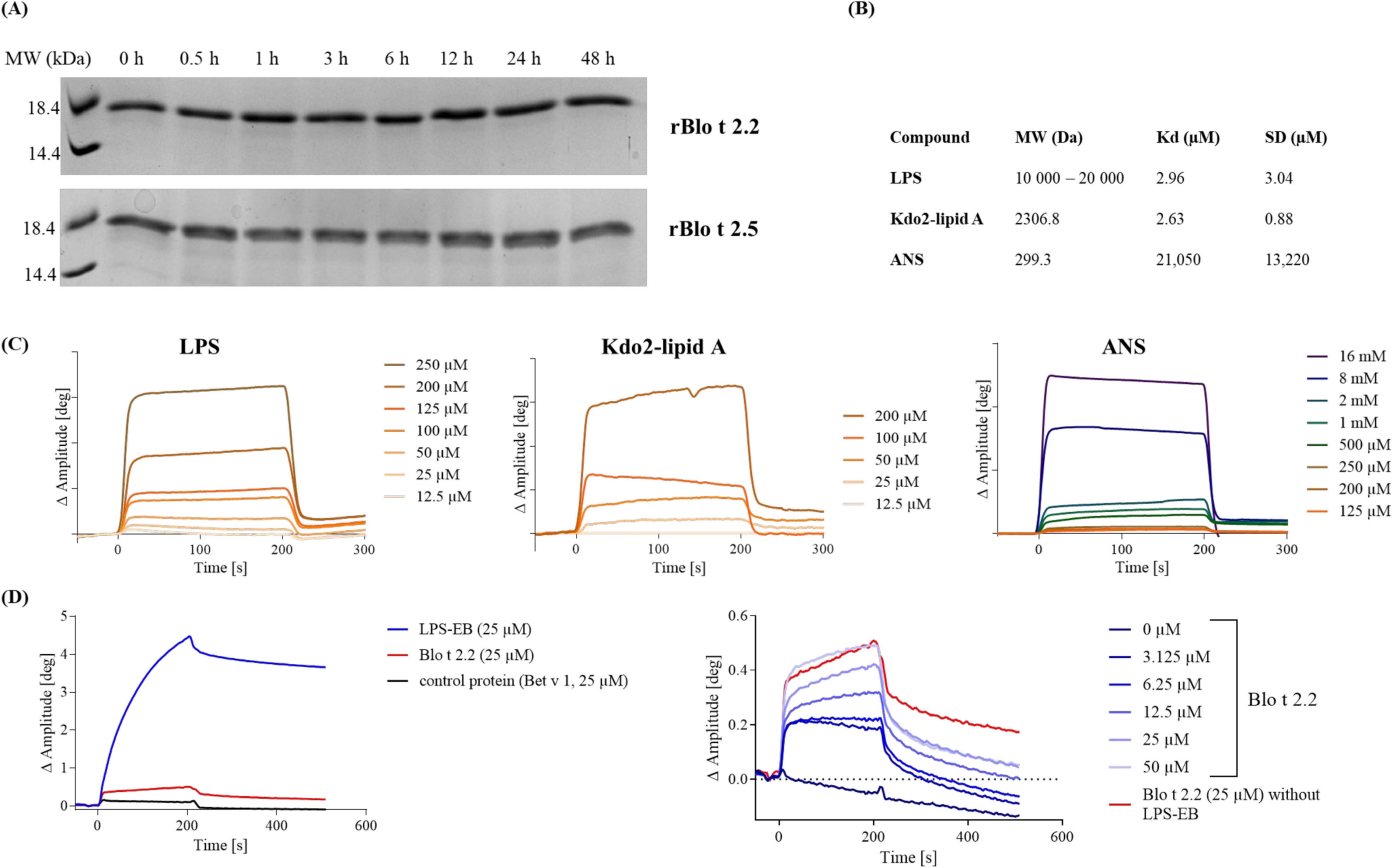
Endolysosomal degradation and LPS-binding activity of rBlo t 2. **(A)** Both rBlo t 2 isoforms underwent proteolytic digestion over 48 h at 37 °C. A 15% SDS-PAGE analysis was performed to visualize the degradation process and stained with Coomassie Blue. **(B)** Table showing the binding affinity (KD) of rBlo t 2.2 to LPS, Kdo2-lipid A and ANS as determined by SAW interaction studies. **(C)** Corresponding binding curves of SAW sensorgram showing the binding interaction between covalently immobilized rBlo t 2.2 and LPS, Kdo2-lipid A and ANS in the free phase. The graphs show the change in amplitude (Δ deg) over time, illustrating the association and dissociation phases of the binding event (on- vs. off-rate). Representative measurements of four independently performed analysis are shown. (**D**, left) Binding of biotinylated LPS to a streptavidin coated SAW sensor chip results in strong binding compared to the weak, unspecific binding observed for Blo t 2.2 and Bet v 1. (**D**, right) The observed dose-dependent interaction between rBlo t 2.2 (0-50 µM) to immobilized LPS (25 µM) occurs due to unspecific binding to the sensor surface (red line).

AAA revealed that the theoretical and experimentally determined amino acid composition matched to a high degree (**Figure 1C**), confirming the integrity of the primary protein sequence. Determination of the secondary structural elements of both isoforms using FTIR spectroscopy revealed a trough at a wavenumber between 1630–1640 cm^-1^ of the second derivative of the Amide I peak, characteristic of antiparallel beta sheet structures (**Figure 1D**). The resistance of the rBlo t 2 isoforms towards endolysosomal degradation was investigated by degradome assay (**Figure 2A**). Even after 48 hours of degradation both isoforms remained intact, reinforcing the strong folding stability of Blo t 2 recombinant isoforms.

To investigate if rBlo t 2.2 exhibits similar LPS-binding activity as described for its homologs Der f 2 and Der p 2, we performed binding assays using SAW technology with covalently immobilized rBlo t 2.2. A low K_D_ was observed for LPS (2.96 ± 3.04 µM) and Kdo2-lipid A (2.63 ± 0.88 µM), while for ANS no relevant binding was observed (21.05 ± 13.22 mM, **Figures 2B, C**). To rule out that the LPS-binding activity obtained for rBlo t 2.2 is not occurring due to unspecific binding to the SAW sensor chip matrix, a second approach was conducted coupling biotinylated LPS to the sensor chip (**Figure 2D**). Coupling of LPS (blue line) resulted in a pronounced phase shift relative to the baseline. In contrast, unspecific binding by rBlo t 2.2 and Bet v 1 was relatively low. By titrating rBlo t 2.2 to immobilized LPS, we obtained a K_D_ of 12.1 µM; however, the highest signal measured with 50 µM rBlo t 2.2 was still lower as that of unspecific binding of 25 µM rBlo t 2.2 to the uncoupled sensor chip matrix. Thus, the LPS-binding activity of rBlo t 2.2 is highly unlikely, as only unspecific binding to the experimental surfaces was measured.

### 3.2 Allergenicity of recombinant Blo t 2 isoforms

**Figure 3:**
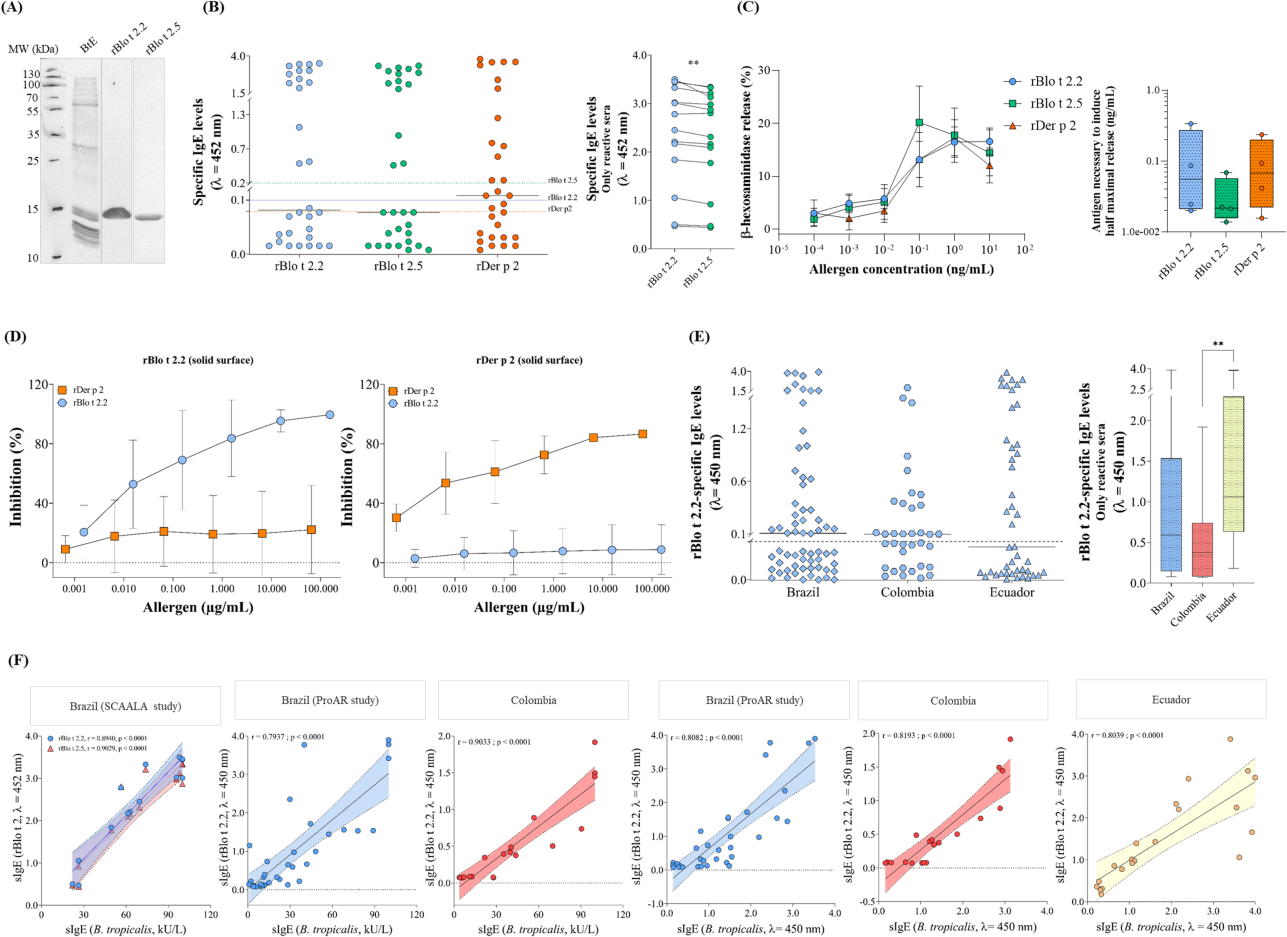
Immunological characterization of rBlo t 2 isoforms. **(A)** Initial Western blot analysis of *B. tropicalis*-reactive Brazilian sera (n = 5, pooled). **(B)** Primary IgE reactivity screening by ELISA (n = 30, SCAALA study; left) and a comparative paired analysis restricted to Blo t 2-reactive sera (**B**, right). Cutoff values are indicated for rBlo t 2.2 (green, 0.155), rBlo t 2.5 (blue, 0.129), and rDer p 2 (red, 0.102). **(C)** Biological activity verified by mediator release assay (n = 4), displayed by antigen concentration (**C**, left) and by determination of half maximum release (**C**, right). **(D)** Evaluation of IgE cross-reactivity via inhibition ELISA (n = 5). (**E**, left) Expanded IgE reactivity screening for rBlo t 2.2 using allergic patient sera from Brazil (n = 64, ProAR study), Colombia (n = 36), and Ecuador (n = 44) and statistical differences among reactive patients (**E**, right). The cut-off line represents an absorbance of 0.075. **(F)** Correlation analyses of rBlo t 2-reactive sera across the different geographic settings. BtE, *Blomia tropicalis* extract. *p < 0.05. **p < 0.01.

Western blot analyses using a sera pool of *B. tropicalis* allergic patients revealed strong IgE reactivity against not only *B. tropicalis* extract, but also both rBlo t 2 isoforms (**Figure 3A**). In ELISA, the rBlo t 2 isoforms bound IgE in around 47% of HDM allergic teenagers compared to 60% responding to rDer p 2 (**Figures 3B**, left graph). The IgE reaction profiles for rBlo t 2.2 and rBlo t 2.5 were identical and four patients were sensitized only to them. In contrast, eight were sensitized only to rDer p 2, and ten sera displayed reactivity to both HDM allergens. Eight samples did not bind to any of the tested recombinant allergens. While rDer p 2 appeared to be more reactive (**Figures 3B**, left graph), no statistically significant differences were observed when the statistical analysis was restricted to reactive sera (data not shown). However, when the comparative paired analysis was restricted to reactive sera, rBlo t 2.2 exhibited significantly higher IgE reactivity than rBlo t 2.5 (**Figures 3B**, right graph).

In the case of huRBL cells passively sensitized with patients’ serum IgE, rBlo t 2.2 and rBlo t 2.5 were able to cause the release of β-hexoaminidase, similar to rDer p 2, without significant differences in the concentrations shown in **Figure 3C**. When calculating the half-maximal release, no significant differences were found to induce the half-maximal mediator release for rBlo t 2.2 in comparison to rBlo t 2.5 and rDer p 2. The mean concentrations of rBlo t 2.2, rBlo t 2.5 and rDer p 2 necessary to trigger the half-maximal mediator release were 1.47 ± 0.20 ng/mL, 1.90 ± 0.18 ng/mL and 1.98 ± 0.55 ng/mL, respectively. As rBlo t 2.2 was more reactive in ELISA experiments, we tested its capacity to inhibit IgE binding to rDer p 2, and vice versa (**Figure 3D**). No evident cross-reactivity with rDer p 2 was observed for rBlo t 2.2 (**Figure 3D**).

Based on the higher allergenicity of rBlo t 2.2, we screened additional samples of *B. tropicalis* allergic patients for IgE reactivity (**Figure 3E**). IgE sensitization to the rBlo t 2.2 isoform was confirmed in 33/64 Brazilian adults (52%), 19/36 Colombian individuals (53%), and 21/44 Ecuadorian children and teenagers (48%). Surprisingly, rBlo t 2.2 reactive sera from Ecuador displayed significantly higher absorbance values than those of Colombian sera, indicating higher IgE levels (**Figure 3E**). Considering all samples, the sensitization rate to rBlo t 2.2 ranged between 47% and 53%, and was on average 50% (87 reactive sera out of 174 *B. tropicalis* allergic patients). Moreover, the IgE reactivity of the rBlo t 2 isoforms strongly correlated with BtE IgE reactivity, from both our in-house extract and the ImmunoCAP^®^ extract (**Figure 3F**) and in all three countries. No significant differences in IgE reactivity by age were observed between the patients (data not shown), suggesting sensitization independently to the age.

### 3.3 rBlo t 2 IgE reactivity and potential association with asthma phenotypes

**Figure 4:**
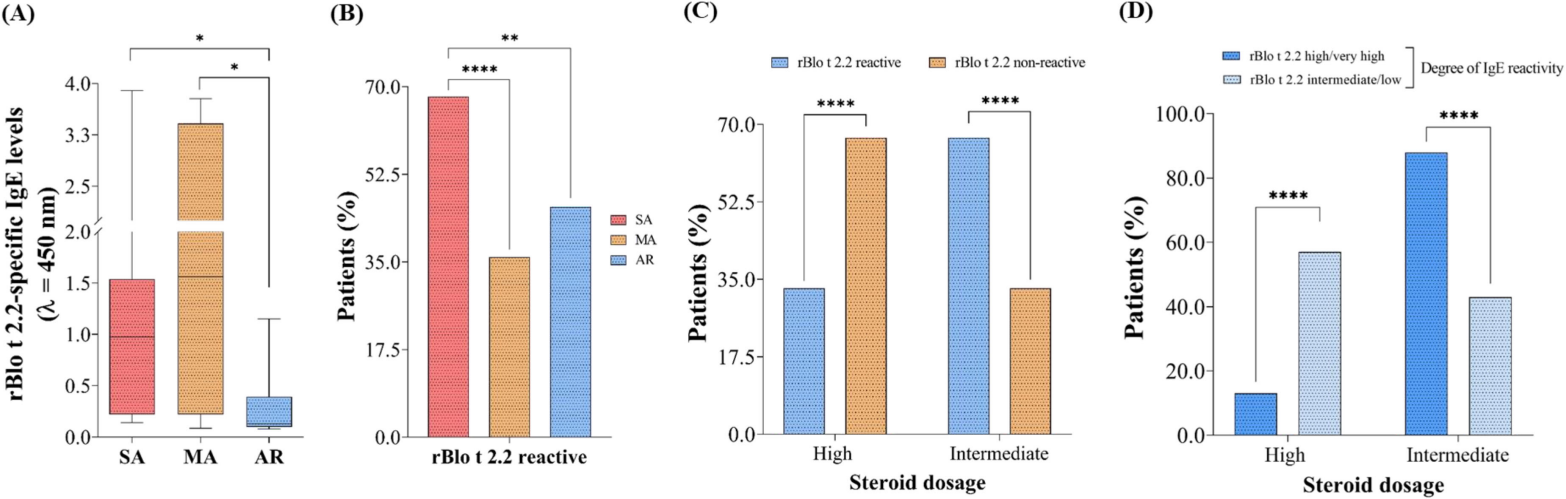
rBlo t 2.2 reactivity in different allergic diseases phenotypes. **(A)** Brazilian sera reactive to *B. tropicalis* were analyzed regarding rBlo t 2.2 IgE reactivity. Only rBlo t 2.2-reactive sera included in the analysis and used to produce the graph. Fisher test was used to verify statistical differences among reactive patients among the phenotypes **(B)**, differences among reactive and non-reactive concerning steroid usage **(C)**, differences among the degree of reactivity and steroid usage **(D)**. SA, severe asthma; MA, mild asthma; AR, allergic rhinitis without asthma. *p < 0.05. **p < 0.01. ****p < 0.0001.

We performed some statistical analyses to address whether the IgE binding features of rBlo t 2.2 would be associated with different allergic disease phenotypes. **Figure 4A** shows that the IgE reactivity was significantly higher in SA and MA patients than those with AR without asthma. In addition, patients with SA had significantly increased sensitization prevalence to rBlo t 2.2 compared to those with MA and AR (**Figure 4B**). Concerning steroid usage related to sensitization to rBlo t 2.2, a significantly higher percentage of non-reactive patients used high dosages of maintenance drugs (**Figure 4C**). In contrast, significantly more reactive patients used the intermediate dosage (**Figure 4C**). **Figure 4D** shows the SA group with a positive reaction to rBlo t 2.2 after categorization by the degree of IgE reaction. High dosage use was more frequent in patients with intermediate/low IgE reactivity, whereas the inverse was observed for patients with high/highly reactive IgE responses to rBlo t 2.2 (**Figure 4D**). These results must be viewed as preliminary indicators of association, given the imbalance between rBlo t 2 reactive and non-reactive samples within the studied groups. The distribution was as follows: SA (15 positive/7 negative), MA (5 positive/9 negative), and AR (13 positive/15 negative).

## 4 Discussion

Group 2 allergens are described as important allergens in several species of dust mites, and they may account for almost 70% of the IgE reactivity of *Dermatophagoides spp* extracts (30, 36, 64, 65). Group 2 allergen of the *B. tropicalis* have several isoforms described at the transcriptome level, but the first test with a recombinant Blo t 2 did not find strong IgE reactivity and the allergen was labeled as a minor one (66). Nevertheless, recent efforts have clearly overturned this notion and Blo t 2 is now considered a mid-tier or major allergen (19, 25–27, 67), considering that it was even the most common sensitizer in asthmatics (13). Moreover, recent studies indicate that sensitization to this allergen is significantly more prevalent in regions where *B. tropicalis* is endemic compared to temperate climate zones (67).

In fact, our research group has found that Blo t 2 was the most present allergenic protein in eleven extracts produced from in-bred lineages of *B. tropicalis* (34). These eleven extracts as well as a commercial extract were analyzed by mass spectrometry, allowing the identification of several Blo t 2 isoforms (34). These findings prompted us to investigate the structural and immunological characteristics of the most abundant variants. However, because isolating individual isoforms from natural extracts with high purity and sufficient yield is technically prohibitive, recombinant expression was employed for the experiments reported herein. Additionally, a previous study comparing recombinant and native Blo t 2 demonstrated that IgE reactivity to rBlo t 2 is highly concordant with the native allergen, suggesting that the recombinant version effectively preserves the essential conformational epitopes (25). Furthermore, as Blo t 2 is a nonglycosylated protein, the use of a prokaryotic expression system such as *E. coli* is unlikely to compromise its structural or immunological integrity. This is corroborated by previous *in vivo* studies where *E. coli*-produced Blo t 2 induced strong allergenic inflammation and biological activity (26). While validating our specific isoforms against the native ones would provide additional confirmation, the limited availability of high-titer patient sera and the technical challenges associated with purifying individual native isoforms in sufficient quantities led us to rely on heterologous expression for the current study.

Our two rBlo t 2 isoforms were structurally stable and allergenic. To date some other recombinant Blo t 2 isoforms have been tested, however, it seems that those were obtained as insoluble, misfolded proteins and displayed low IgE reactivity (25, 66), whereas the one acquired in the soluble fraction exhibited a higher IgE reactivity (13, 26), comparable to our results. On the other hand, the observed differences might result from the sIgE binding features of various isoforms of the same allergen, which has been reported for several allergens e.g. one isoform of the birch pollen allergen Bet v 1 (Bet v 1d) is a hypoallergen (38, 68, 69).

Another important factor, that may be related to allergenicity, is the resistance towards endolyssomal degradation. Both isoforms did not degrade at all within 48 h, similar to what was described for rDer p 2 (36). Although there was no noticeable difference between the two isoforms in this aspect, rBlo t 2.2 still seems to be more allergenic than rBlo t 2.5 in the ELISA for the SCAALA study. It has been previously hypothesized that either low or excessively strong proteolytic stability of allergens may lead to low peptide abundance in the late endosome and, in turn, induce higher allergenic responses (42, 70). Major allergens of *B. tropicalis* have behaved differently, degrading faster *in vitro* (40, 41) and inducing inflammation *in vivo* (40, 71). Although classified in the present study as a mid-tier to major allergen, recent investigations involving a different rBlo t 2 isoform have demonstrated that this *B. tropicalis* allergen effectively induces inflammatory responses *in vivo* (26).

Regarding the LPS-binding activity of rBlo t 2.2, we could not confirm that the allergen is binding LPS. Both HDM allergens, Der f 2 and Der p 2, were previously shown to bind LPS, and, thus, this binding activity of the Niemann-Pick C2 allergen family was interpreted as an adjuvant activity of these major allergens by mimicking MD-2 and facilitating TLR4 activation leading to allergic sensitization in mice (72–74). Recently, both, the LPS-binding activity as well as the MD-2 mimicry of HDM allergens, have been questioned (75). Der p 2 lacks critical key residues of MD-2 for TLR4 binding, such as the DDD motif (amino acid residues 99-101), and most of the hydrogen bonds and salt bridges required for the TLR4-MD-2 interaction cannot be formed by Der p 2, as sequence alignments indicate. MD-2 binds to TLR4 with a dissociation constant of 0.8 nM, and with 3 nM to LPS (76). In comparison, the K_D_ values for rBlo t 2.2 binding to LPS obtained in our experiments were more than 1000-fold higher and, based on our experiments with the streptavidin sensor chip, mainly occurred due to unspecific binding to the sensor chip matrix. Additionally, these findings align with prior studies indicating that Blo t 2 isoforms may have impaired lipid-binding capacity. All Blo t 2 variants lack the key TLR4-binding residue K77 and lack the aromatic residue at position 75 – equivalent to Tyr102 in MD-2 – which is critical for mediating TLR4-complex formation (77, 78). Of note, a positive control antigen such as MD-2, was not included in the SAW experiments, which would have further strengthened our results and represents a limitation of our study. Future studies should reevaluate the LPS binding potential of Der p 2 and should address the TLR4-associated innate immune activation potential of Der p 2 and Blo t 2 using TLR4 reporter assays and dendritic cell-based models.

Anti-Blo t 2 IgE reactivity that was reported herein is indicative of true sensitization to this allergen, as there was no evidence of relevant cross-reactivity with Der p 2. This outcome suggests that the produced rBlo t 2-sIgE is a consequence of primary sensitization to *B. tropicalis* and not a result of cross-reactivity to Der p 2. Considering that the two allergens share less than 40% of sequence similarity (12), our results were expected and are in agreement with previous reports on other Blo t 2 isoforms (25, 26). Indeed, these previous studies focused on other Blo t 2 isoforms have reported low inhibition percentages (< 20%) and estimated that Blo t 2 may possess 20–50% unique IgE epitopes compared to its *D. pteronyssinus* counterparts (25, 26). Our results align with these observations suggesting a significant molecular divergence in the IgE-binding surfaces of these allergens. Nevertheless, to rigorously confirm these preliminary findings, cross-reactivity should be evaluated using a larger cohort and a more extensive experimental approach, such as the methodology previously described by Kuo et al. (79).

Interestingly, the IgE reactivity to rBlo t 2.2 observed in the Ecuadorian cohort was significantly higher than that observed in Colombia. While the difference in sample sizes between these cohorts may influence this statistical observation, prior studies have established that geographical variations can significantly impact sensitization patterns (16, 67, 80, 81), as recently corroborated by our group (82). Environmental factors associated with varying climates and altitudes likely dictate the concentration and distribution of specific mite species and their isoforms (68, 83–86). Consequently, populations exposed to distinct allergen profiles or varying exposure gradients may develop disparate sensitization frequencies and intensities. Therefore, our findings highlight the need for further standardized environmental monitoring to confirm whether these variations in Blo t 2 reactivity reflect stable regional immunological trends or transient environmental fluctuations.

Correlation analysis indicated a strong positive correlation between rBlo t 2 and BtE IgE responses. Regardless of the origin of various extracts of *B. tropicalis*, some isoforms of Blo t 2 were always present and, in many cases, represented the most abundant allergen in the extract (26, 34). Consequently, the correlation findings underscore the necessity of incorporating our two Blo t 2 isoforms (especially rBlo t 2.2) into diagnostic panels for HDM allergy. One could suggest that the high abundance of these allergens likely leads to increased environmental exposure, facilitating a higher likelihood of IgE sensitization. However, there are no published data that could confirm a direct relationship between high presence of an allergen in extracts or fecal pellets and higher sensitization. Indeed, rBlo t 5 remains the major allergen of *B. tropicalis*, even though it is, sometimes, present in small quantities in extracts (14, 34, 40, 52, 87). In fact, our data may emphasize that IgE responses are, on occasion, inversely correlated with high concentrations of HDM allergens in the extract and, consequently, in the environment (25, 87, 88).

Interestingly, during our initial IgE reactivity screening with sera from the SCAALA study, we observed statistical differences between the isoforms. Reginald, Pang & Chew (2019) described by in silico analysis that the amino acid residues G23, H29, and I96 are implicated in forming critical conformational epitopes (25). While most residues are conserved across the Blo t 2 isoforms characterized here, we identified key substitutions at positions 96 and 100. Specifically, Blo t 2.2 possesses a valine instead of an isoleucine at position 96, and a valine instead of a leucine at position 100. Although valine, isoleucine, and leucine are all aliphatic, hydrophobic amino acids, these substitutions in Blo t 2.2 may subtly alter the protein’s surface topography. Such changes could potentially increase the accessibility of specific epitopes or optimize hydrophobic interactions within the IgE-binding region, thereby explaining the enhanced IgE reactivity observed for this isoform. This is consistent with findings in other allergens where minor amino acid substitutions in hydrophobic residues significantly altered IgE recognition (89–93). However, future studies producing mutants with alanine substitutions, for instance, will be important to determine if these two valine substitutions are truly the key residues responsible for the higher IgE reactivity observed for Blo t 2.2. Nonetheless, this observation does not imply that Blo t 2.5 lacks clinical relevance. On the contrary, additional multicenter studies encompassing all five Blo t 2 isoforms currently recognized by the IUIS are warranted, particularly as several have displayed distinct IgE reactivity patterns in previous reports (13, 16, 25, 26, 66, 67).

A recent study has found that the major allergens Blo t 5 and Blo t 21 were associated with asthma, but statistical evidence was not found for other *B. tropicalis* allergens, even though the most frequent sensitizer among Colombian patients was rBlo t 2 (13). In contrast, our data suggest rBlo t 2.2 IgE responsiveness is a potential biomarker for SA in Brazil. These findings were similar to previous studies associating Der p 2 sensitization with asthma and its severe phenotype (94, 95). Other studies have associated IgE reactivity against specific allergens with the severity of some allergic diseases (63, 96, 97). Additionally, our data indicate that steroid use and dosage in SA patients may influence the degree of IgE reactivity. Similar findings were reported before, including a down-modulation of mite sIgE (98–100). The high prevalence of rBlo t 2.2-reactive sera in SA, on the other hand, in patients receiving an intermediate corticosteroid dosage confirms that pharmacotherapy does not consistently result in a reduction of sIgE, although it suppresses airway inflammation, as reported in some studies (101–103). Nevertheless, additional comprehensive investigation is required to further elucidate the functional role of Blo t 2 in the onset and progression of SA associated with *B. tropicalis* allergy. The clinical findings of this study warrant validation through future research utilizing larger, well-powered cohorts. We acknowledge that the limited sample size within specific asthma phenotypes represents a constraint of our current investigation. Future studies should address the association with severe asthma by employing multivariate models adjusted for potential confounders, such as age, sex, and total IgE levels. Such rigorous analysis is essential to confirm our preliminary findings and definitively establish whether Blo t 2.2 serves as a robust sensitization biomarker for severe asthma.

Our detailed physicochemical and immunological characterization demonstrates the utility of a highly stable, easy to purify and soluble recombinant Blo t 2 isoforms. Given its differential sensitization patterns among populations of Brazil, Colombia and Ecuador and its potential as a sensitization biomarker for severe asthma in Brazilian adults, we strongly recommend the inclusion of rBlo t 2 in molecular diagnostic panels, which are currently becoming a trend for allergy diagnosis, as suggested by recent studies (16, 67). Nevertheless, our findings need validation by future research in larger and longitudinal cohorts, probably using a multi-allergen panel. Furthermore, this isoform serves as a potential backbone for the construction of hypoallergenic mutants for use in allergen-specific immunotherapy.

## Data Availability

The original contributions presented in the study are included in the article/supplementary material. Further inquiries can be directed to the corresponding authors.
